# Effect of Food Migrations of PEEK-Modified Atmosphere Packaging Materials on Mitochondrial Damage via PGC-1α/Nrf2 Signaling Pathway

**DOI:** 10.3390/toxics13121054

**Published:** 2025-12-05

**Authors:** Sihui Guo, Kaile Li, Wei Li, Hao Huang, Yalan Zhang, Qinwen Zhou, Qi He, Zhini He, Weiliang Wu, Xingfen Yang, Qinzhi Wei

**Affiliations:** Food Safety and Health Research Center, Guangdong Provincial Key Laboratory of Tropical Disease Research, School of Public Health, Southern Medical University, Guangzhou 510515, China; gsh79190624@163.com (S.G.); lkl5454han@163.com (K.L.); lwliweiyy@163.com (W.L.); hh4240123@163.com (H.H.); zhangyalan0616@163.com (Y.Z.); qwzhouz@163.com (Q.Z.); heqi@163.com (Q.H.); hezhinihzn@126.com (Z.H.); wu1108@smu.edu.cn (W.W.)

**Keywords:** PEEK, PGC-1α/Nrf2, mitochondrial damage

## Abstract

Poly Ether-Ether Ketone (PEEK) is used in modified atmosphere packaging (MAP) for fruit and vegetable preservation, but raises health concerns. This study investigated the effects of PEEK food migrations on liver cell mitochondrial damage. Food simulants (95% ethanol, 10% ethanol, and 4% acetic acid) were used for migration tests according to guideline recommendations, and liver cells were treated with PEEK food migrations for 24 h. Results showed decreased cell viability, increased reactive oxygen species (ROS), reduced mitochondrial membrane potential (MMP), mitochondrial DNA copy number (mtDNAcn), and down-regulated PGC-1α/Nrf2 pathway-related genes (Sirt1, PGC-1α, NRF1, Nrf2, TFAM). Furthermore, these alterations were reversed, and mitochondrial damage was alleviated by the addition of the PGC-1α activator ZLN005. In conclusion, high PEEK concentrations induce mitochondrial toxicity in liver cells via the PGC-1α/Nrf2 pathway, posing health risks and necessitating safe dosage limits in food packaging.

## 1. Introduction

Poly Ether-Ether Ketone (PEEK) is a high-performance aromatic thermoplastic renowned for its hydrolysis resistance, high-temperature stability, abrasion resistance, strength, and dimensional stability. These properties enable its widespread use in the medical, semiconductor, and food industries. Sulfonation modification of PEEK significantly enhances its affinity for CO_2_, improves gas permeability., making it suitable for fruit and vegetable preservation [1]. Additionally, its excellent chemical stability and mechanical properties protect against external damage, making PEEK increasingly attractive for developing food packaging materials. It has been demonstrated that the use of modified PEEK for air-conditioned packaging is effective in maintaining the quality of fruits and vegetables, such as sundried fruits [2] and chili peppers [3]. In the early stages, the research group developed a modified atmosphere packaging (MAP) material using PEEK as the raw material, which is well-suited for preserving fruits and vegetables under high-temperature and humidity climate conditions of southern China.

In recent years, there has been a great deal of concern about the migration of food packaging materials into food and the potential health risks for humans [4]. Current research on PEEK-modified materials for MAP is increasing, and investigations into the safety profiles of food contact migrants derived from these materials remain critically underexplored. Therefore, further investigation is required to clarify the health risks associated with migrants from PEEK-based MAP materials, providing a scientific basis for hazard assessment and regulating safe application of PEEK materials.

In 2007, the US National Research Council (NRC) elaborated the Toxicity Testing in the 21st Century (TT21C), which proposed for the first time the establishment of an in vitro toxicity-testing method based on the ‘toxicity pathway’ and ‘toxicity mechanism of action’ [5]. Linking key toxicity pathway molecules to their biological effects establishes dose–response models, enhancing testing efficiency and reducing animal use [6]. Currently, multiple regions, including China, the EU, the US, and Japan, have established animal welfare regulations. The adverse outcome pathway (AOP) framework connects molecular initiating event (MIE)) to adverse outcome (AO) through key events (KEs), enabling prediction of high-level toxicity from low-level events [7]. By establishing AOPs, the number of tests, times, and costs will be reduced, while animal welfare will be further safeguarded based on the accuracy and reliability of compound toxicity assessments [8].

The liver is the major organ responsible for the metabolism of substances and energy in the body. L-02 cells are derived from human hepatocytes and are widely used to assess health risks and toxicity mechanisms in vitro [9]. Exogenous chemicals endanger health mainly through oxidative stress, with mitochondria, the primary energy producers, as critical targets [10]. AOP-Wiki and literature reviews identify mitochondrial dysfunction and increased ROS as frequent MIEs of mitochondrial toxicity AOPs [11]. Downstream KEs, such as mtDNA damage, ATP reduction, oxidative stress, and MMP loss, culminate in AOs, including liver injury [12]. Central to this process, the PGC-1α/Nrf2 pathway involves Sirtuin 1 (Sirt1) upregulating peroxisome proliferator-activated receptor gamma coactivator-1α (PGC-1α) via deacetylation. This master regulator of mitochondrial biogenesis, once activated through post-translational modification or transcriptional upregulation, controls the expression of mitochondrial proteins and drives mitochondrial biogenesis [13]. PGC-1α, in turn, activates nuclear factor erythroid 2-related factor 2 (Nrf2), which not only supports antioxidant defenses but also acts as a transcriptional modulator of mitochondrial biogenesis. Specifically, Nrf2 can directly induce NRF1 (nuclear respiratory factor 1) by binding to ARE sequences in the NRF1 gene promoter, thereby promoting NRF1 expression [14]. Elevated NRF1 subsequently increases the expression of mitochondrial transcription factor A (TFAM), which is essential for mtDNA transcription and replication [15]. Moreover, Nrf2 itself is required for the full induction of mitochondrial biogenesis programs—including NRF1 and TFAM expression—during metabolic or oxidative stress [16]. Through this cascade, PGC-1α-driven Nrf2 activation stimulates NRF1/TFAM signaling, thus facilitating mitochondrial biogenesis and ATP synthesis. Additionally, PGC-1α could also directly regulate UCP2 and MnSOD expression, modulating cellular antioxidant defenses [17]. Collectively, the PGC-1α/Nrf2 pathway represents the central regulatory pathway counteracting oxidative damage by synchronously orchestrating mitochondrial biogenesis, ROS scavenging, and energy homeostasis, making it a critical mechanistic focus for assessing xenobiotic-induced hepatotoxicity.

This study is conducted within the framework of the adverse outcome pathway (AOP). We investigate mitochondrial impairment caused by PEEK migrations and identify key toxicity pathways associated with mitochondrial dysfunction, with emphasis on the PGC-1α/Nrf2 signaling axis. The results will provide scientific evidence and a theoretical foundation for hazard assessment and the development of safety standards for PEEK air-conditioning packaging films previously developed by our research group.

## 2. Materials and Methods

### 2.1. Materials and Reagents

RPMI-1640, 0.25% Trypsin, and fetal bovine serum (FBS) were purchased from Gibco (Grand Island, NY, USA). PEEK was purchased from Shanghai Yingcai Industrial Co., Ltd. (Shanghai, China). Anhydrous ethanol (HPLC), acetic acid (HPLC), dimethyl sulfoxide, and MTT were purchased from Sigma-Aldrich (St. Louis, MO, USA). ZLN005 was purchased from MedChemExpress (Monmouth Junction, NJ, USA). Antibodies against GAPDH (5174S), Nrf2 (12721), UCP2 (89326), and MnSOD (13141) were purchased from Cell Signaling Technology (Danvers, MA, USA). Antibodies against PGC-1α (ab191838), NRF1 (ab175932), and TFAM (ab176558) were purchased from Abcam (Cambridge, MA, USA). Antibodies against sirt1 (13161-1-AP) were purchased from Proteintech (Wuhan, China). Horseradish peroxidase (HRP)-conjugated goat anti-rabbit IgG (111-036-045) was purchased from Jackson ImmunoResearch Laboratories (West Grove, PA, USA). Phosphate-buffered saline (PBS) and skimmed milk were purchased from Pythonbio (Guangzhou, China).

### 2.2. Cell Culture

L-02 cells for the experiments were donated by Liu Qizhan Research Group of Nanjing Medical University and obtained from the Institute of Cell Biology, Shanghai Institutes for Life Sciences, The Chinese Academy of Sciences. It was confirmed that L-02 cells were not contaminated by HeLa cells to ensure the reliability of this paper (Supplementary Figure S1). The cells were cultured in RPMI-1640 medium supplemented with 10% FBS and 1% penicillin at 37 °C with 5% CO_2_ in a CO_2_ incubator (Thermo Fisher Scientific Heracell™ VIOS 160i, Waltham, MA, USA). The medium was changed every 2 days. When the cells reached 80–90% confluence, they were washed twice with PBS, then trypsinized and centrifuged at 1000 rpm for 3 min, and then the cells were passed at a ratio of 1:2–1:3.

### 2.3. Preparation of PEEK Food Migrations

According to the guidelines GB 31604.1-2015 National Standard for Food Safety General Principles for migrations Tests of Food Contact Materials and Products, and taking into account the intended use of the PEEK membrane, three food simulants were selected: 95% (*v*/*v*) ethanol to simulate fatty foods, 10% (*v*/*v*) ethanol to simulate aqueous foods, and 4% (*v*/*v*) acetic acid to simulate acidic foods. Food simulants were prepared volumetrically: 95% (*v*/*v*) ethanol by diluting 50 mL ultrapure water to 1 L with ethanol (fatty food simulants); 10% (*v*/*v*) ethanol by diluting 100 mL ethanol to 1 L with ultrapure water (aqueous food simulants); and 4% (*v*/*v*) acetic acid by diluting 40 mL acetic acid to 1 L with ultrapure water (acidic food simulants). Standard preparation protocols are documented in Supplementary Materials Text S1. PEEK membranes were cut into 30 cm^2^ pieces, ultrasonically cleaned in ultrapure water, dried for 30 min, and weighed. Five membrane specimens were fully submerged in 250 mL food simulants and in Constant Temp. Water Bath (Shanghai Yiheng Instruments DK-8D, Shanghai, China), maintained at 40 °C for 10 days to simulate actual food contact duration and temperature. Post-migration, simulants were concentrated to approximately 10 mL via rotary evaporation (IKA Works RV 10, Staufen, Germany), followed by complete solvent removal through nitrogen purging (Beijing Hengao Tech HAD-24, Beijing, China). The residues were reconstituted in DMSO to obtain food migrations. Further experimental details are provided in the Supplementary Materials Text S2. The row experimental record of the migration test for PEEK Food Contact Material is presented in the Supplementary Table S2. UPLC-MS/MS confirmed batch consistency (chromatographic conditions: Amide column, mobile phase is 0.1% formic acid water + 5 mmol/L ammonium acetate (Phase A) and acetonitrile (Phase B), flow rate: 0.20 mL/min, injection volume: 2 μL), enabling preparation of a 4000 mg/mL migration stock solution for subsequent investigations. More details of the protocols are presented in the Supplementary Materials Text S3. Analysis of the base peak chromatograms obtained from UPLC-MS/MS characterization of PEEK food migrations is presented in the Supplementary Figure S2. Additional analyses are presented in the Supplementary Materials Text S4.

### 2.4. Cell Viability Assays

L-02 cells in logarithmic phase and in good condition were seeded into 96-well culture plates at a density of 5000 cells per well. After incubation for 24 h, the growth medium was replaced with various concentrations of PEEK oily, water-based, and acidic food migrations (0, 1, 2.5, 5, 10, 20, and 40 mg/mL) incubated for an additional 24 h. Meanwhile, the cells were treated with different concentrations of DMSO as a solvent control. After treatment, the cells were exposed to 0.5 mg/mL MTT for 4 h, then the culture medium was discarded carefully, and 150 μL DMSO was added to dissolve the formazan crystals, which were shaken continuously for 10 min. Absorbance at wave 490 nm was measured using a microplate reader (BioTek Instruments Synergy H1, Winooski, VT, USA).

### 2.5. Transmission Electron Microscopy (TEM) for Analysis of Mitochondria

L-02 cells in good condition were seeded into 6-well culture plates at a density of 5 × 10^5^ cells per well. After incubation for 24 h, the growth medium was renewed with various concentrations of PEEK oily and water-based food migrations (40 mg/mL) and PEEK acidic food migrations (20 mg/mL) for 24 h. Cells cultured in medium without PEEK food migrations for 24 h served as the control. After discarding the culture medium, the cells were washed twice with PBS before being fixed in 2.5% glutaraldehyde. The samples were then fixed with osmic acid (OsO_4_), embedded in Epon812, and sectioned into ultrathin slices. These sections were stained with uranyl acetate and lead citrate, and mitochondrial structures were observed using TEM (Hitachi H-7500, Tokyo, Japan).

### 2.6. Intracellular ATP Analysis

L-02 cells in logarithmic phase and in good condition were seeded into 6-well culture plates at a density of 5 × 10^5^ cells per well, and after a 24 h incubation, the growth medium was renewed with various concentrations of PEEK oily and water-based food migrations (0, 2.5, 10, and 40 mg/mL) and PEEK acidic food migrations (0, 1, 5, and 20 mg/mL) for 24 h. Cells in complete medium served as the zero-dose control group. After incubation for 24 h, the cells were washed twice with PBS and lysed in 200 μL ATP lysis solution per well for 15 min (Beyotime, Haimen, China). The lysates were centrifuged at 12,000× *g* for 5 min at 4 °C, and the supernatants were collected. The ATP concentration corresponding to the relative luminescence unit (RLU) of the samples was derived from the ATP standard curve, and the final ATP content was normalized to the protein concentration.

### 2.7. Measurement of Cytosolic Reactive Oxygen Species (ROS)

ROS were measured with the DCFH-DA probe (Nanjing Jiancheng Bioengineering Institute, Nanjing, China). A negative control tube contained cells in medium without the probe, while a positive control tube contained cells treated with the probe and supplemented with a ROS inducer. After a 24 h infection period, the culture medium was discarded, and the cells were washed with PBS. All experimental groups (including treatment groups and the zero-dose control group) were then incubated with 10 μM DCFH-DA for 30 min at 37 °C, followed by two PBS washes. The cells were trypsinized, centrifuged at 1000× *g* for 3 min, washed twice with PBS, and then collected and transferred to flow sampling tubes. The fluorescence intensity of the cells was detected by flow cytometry using 488 nm excitation and 525 nm emission wavelengths.

### 2.8. Detection of Mitochondrial Membrane Potential (MMP)

MMP was measured with the JC-1 probe (KEYGEN BIOTECH, Nanjing, China). Following a 24 h infection period, the culture medium was discarded, and the cells were washed twice with PBS. All experimental groups, including treatment groups and zero-dose control, were then incubated with 1 mL of JC-1 working solution for 20 min at 37 °C in the dark. After two washes with JC-1 incubation buffer, cells were collected into flow cytometry tubes. JC-1 exhibits concentration-dependent fluorescence: forming green-fluorescent monomers at low concentrations and red-fluorescent J-aggregates at high concentrations. In mitochondria with polarized Δψm, JC-1 accumulates and forms J-aggregates, while depolarization promotes monomer formation. MMP was assessed by calculating the ratio of red fluorescence intensity (aggregate) to green fluorescence intensity (monomer) (red/green ratio). Monomers emit green fluorescence detected through the FITC channel (FL1, emission 530 nm), while J-aggregates emit red fluorescence measured via the PE channel (FL2, emission 590 nm). Fluorescence intensity was quantified by flow cytometry at 488 nm excitation.

### 2.9. Analysis of Mitochondrial DNA Copy Number (mtDNAcn)

L-02 cells in logarithmic phase and in good condition were seeded into 12-well culture plates at a density of 2 × 10^5^ cells per well. After incubation for 24 h, the growth medium was replaced with various concentrations of PEEK oily and water-based food migrations (0, 2.5, 10, and 40 mg/mL) and PEEK acidic food migrations (0, 1, 5, and 20 mg/mL) for 24 h. As a control, cells were cultured in medium containing no PEEK food migrations for 24 h. Total DNA was extracted from cells using a universal genomics DNA kit (TIANGEN, Beijing, China), and 10 ng of DNA was used for qPCR analysis. ND1 was the target gene, with HBG (human β-globin protein) serving as the internal reference gene. Primer sequences for ND1 and HBG are provided in Supplementary Table S1. Samples were cycled 40 times at 95 °C for 5 s and 64 °C for 34 s, and results were calculated using 2^−ΔΔCT^.

### 2.10. Western Blot Analysis

L-02 cells in logarithmic phase and in good condition were seeded into 6-well culture plates at a density of 5 × 10^5^ cells per well. After incubation for 24 h, the growth medium was replaced with various concentrations of PEEK oily and water-based food migrations (0, 2.5, 10, and 40 mg/mL) and PEEK acidic food migrations (0, 1, 5, and 20 mg/mL) for 24 h. The control group comprised cells cultured for 24 h in medium lacking PEEK food migrations. Total cellular protein was extracted using a BCA protein assay kit (KEYGEN BIOTECH, Nanjing, China). Protein samples (40 μg) were separated by 10–12% SDS-PAGE and transferred to polyvinylidene fluoride (PVDF) membranes by gel electrophoresis (Bio-Rad, Hercules, CA, USA). The membranes were blocked with 5% skim milk and then incubated with primary antibodies against Sirt1 (1:3000), PGC-1α (1:1000), Nrf2 (1:1000), NRF1 (1:5000), TFAM (1:10,000), UCP2 (1:1000), MnSOD (1:1000), and GAPDH (1:1000) overnight with gentle agitation at 4 °C. After three washes with TBST, the membranes were incubated with goat anti-rabbit secondary antibodies (dilution 1:5000) for 90 min at room temperature. Protein expression was quantified using an electrochemiluminescence (ECL) detection system (Tanon Science & Technology Co., Ltd., Shanghai, China), and the final protein bands were analyzed using Image J software version 1.54g (NIH, Bethesda, MD, USA). The results are expressed as the optical density value of the target protein divided by the optical density value of the internal reference protein.

### 2.11. Statistical Analysis

All measurements were obtained from at least 3 independent experiments. Statistical analysis was performed using Graph Pad Prism version 9.0 (GraphPad Software, San Diego, CA, USA). Data were first tested for normality using the Shapiro–Wilk test. For normally distributed data, one-way ANOVA was applied, followed by Tukey’s post hoc test for multiple comparisons. Data are presented as mean ± SD, and differences were considered statistically significant at *p* < 0.05.

## 3. Results

### 3.1. PEEK Food Migrations Affect the Viability of L-02 Cells

**Figure 1:**
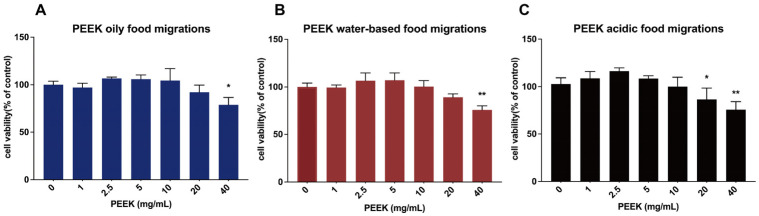
Effect of different concentrations of PEEK food migrations on the viability of L-02 Cells. The viability of L-02 cells was detected by MTT assay. (**A**) Cells were treated with PEEK oily food migrations (0, 1, 2.5, 5, 10, 20, and 40 mg/mL) for 24 h. (**B**) Cells were treated with PEEK water-based food migrations (0, 1, 2.5, 5, 10, 20, and 40 mg/mL) for 24 h. (**C**) Cells were treated with PEEK acidic food migrations (0, 1, 2.5, 5, 10, 20, and 40 mg/mL) for 24 h. Data are expressed as the mean ± standard deviation (*n* = 4). * *p* < 0.05, ** *p* < 0.01: compared to control cells cultured in the absence of PEEK food migrations.

The MTT assay results indicated that cell viability decreased significantly (*p* < 0.05) with oily and water-based food migrations at PEEK 40 mg/mL, and acidic food migrations at 20 mg/mL (Figure 1A–C). Based on these results, we selected the following concentrations for subsequent experiments: 0, 2.5, 10, and 40 mg/mL for oily and water-based PEEK food migrations, and 0, 1, 5, and 20 mg/mL for acidic PEEK food migrations.

### 3.2. PEEK Food Migrations Damage Mitochondrial Structure in L-02 Cells

**Figure 2:**
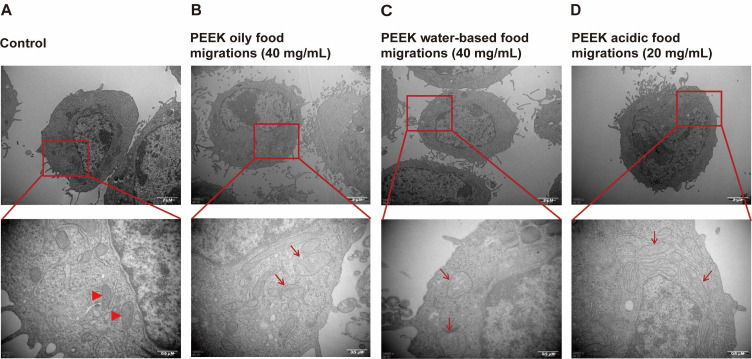
Effect of mitochondrial structure from PEEK food migrations in L-02 cells. Cells were treated with PEEK food migrations for 24 h. The mitochondrial structure was observed by TEM. The top images were observed at 10,000× magnification, the bottom images were observed at 40,000× magnification, and triangles and arrows indicate mitochondria. (**A**) Control. (**B**) 40 mg/mL of PEEK oily food migrations. (**C**) 40 mg/mL of PEEK water-based food migrations. (**D**) 20 mg/mL of PEEK acidic food migrations.

Based on the cell viability results, we observed the mitochondrial structure after treatment with PEEK food migrations using TEM. Compared to the control group, the mitochondrial structure was obviously damaged after treatment with PEEK food migrations for 24 h in L-02 cells, including mitochondria swelling with significant expansion of the organellar matrix, along with cristae disarray and loss, where the characteristic lamellar cristae structures were fragmented, shortened, or entirely obliterated, compromising the inner membrane architecture. Additionally, vacuolar degeneration was observed as evident as clear, membrane-bound vacuoles within the mitochondrial matrix, indicative of severe structural disintegration (Figure 2A–D).

### 3.3. PEEK Food Migrations Injure Mitochondrial Function in L-02 Cells

**Figure 3:**
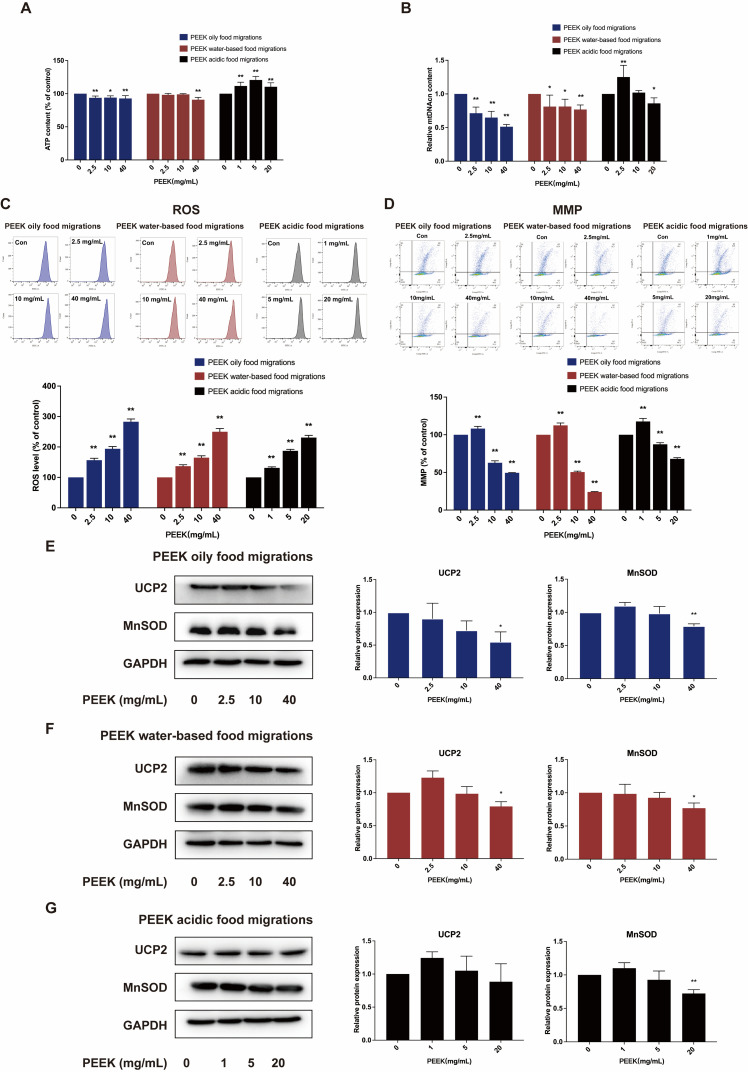
Effect of PEEK food migrations on mitochondrial function in L-02 cells. Cells were treated with PEEK food migrations for 24 h. (**A**) ATP concentrations were detected by chemical luminescence. (**B**) The content of mtDNAcn was measured by qPCR. (**C**) ROS levels were measured with the DCFH-DA probe. (**D**) MMP levels were measured with the JC-1 probe. (**E**–**G**) The relative expressions of UCP2 and MnSOD were assessed by Western Blot analysis. Data are expressed as the mean ± standard deviation (*n* = 3). * *p* < 0.05, ** *p* < 0.01: compared to control cells cultured in the absence of PEEK food migrations.

The results revealed that the PEEK migrations at concentrations of 2.5–40 mg/mL in oily food and 40 mg/mL in water-based food significantly decreased cytosolic ATP levels (*p* < 0.05). Conversely, PEEK migrations at concentrations of 1–20 mg/mL in acidic food significantly increased ATP levels (*p* < 0.05) (Figure 3A). Additionally, PEEK migrations at 2.5–40 mg/mL in oily and water-based food migrations significantly reduced mtDNAcn in a dose-dependent manner (*p* < 0.05). Notably, PEEK migrations at 20 mg/mL in acidic food also caused a significant decrease in mtDNAcn (*p* < 0.05) (Figure 3B). Flow cytometry analysis showed that PEEK migrations in oily, water-based, and acidic food significantly elevated ROS levels (*p* < 0.05) (Figure 3C). Furthermore, mitochondrial membrane potential (MMP), a crucial parameter of mitochondrial function, was significantly decreased as measured by flow cytometry. The response to PEEK concentration exhibited a biphasic pattern, with an increase in concentration up to 2.5 mg/mL in oily and water-based food migrations, and up to 1 mg/mL in acidic food migrations, followed by a decrease at higher concentrations (*p* < 0.05) (Figure 3D). Considering the changes in ATP, mtDNAcn, ROS, and MMP after treatment by PEEK food migrations, we further detected the expression of mitochondrial function-related proteins using Western Blot analysis. Immunoblot analysis indicated that the protein expression levels of UCP2 and MnSOD do not affect cell viability significantly at low concentrations of PEEK, only at PEEK concentrations of 40 mg/mL in oily and water-based food migrations, and 20 mg/mL in acidic food migrations, protein expression levels of UCP2 and MnSOD could be down-regulated (Figure 3E–G). These results suggested that food migrations can cause mitochondrial function injury in L-02 cells at high concentrations of PEEK.

### 3.4. PEEK Food Migrations Inhibit the Signaling Pathway of Mitochondrial Damage

**Figure 4:**
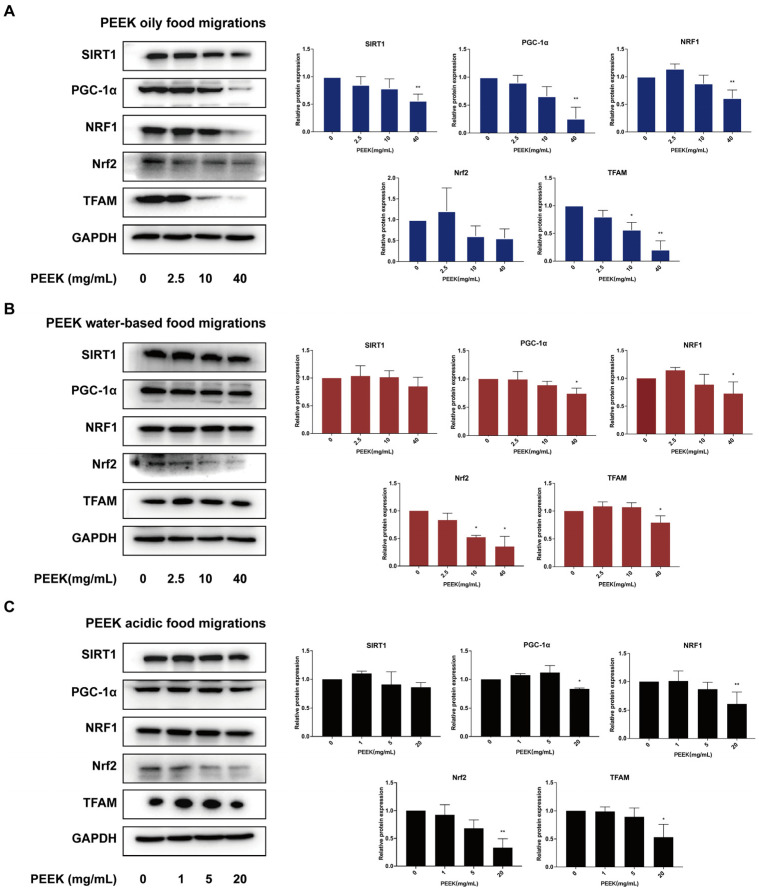
Effect of PGC-1α/Nrf2 signaling pathway by PEEK food migrations on L-02 cells. Cells were treated with PEEK food migrations for 24 h. Western Blot analyses were performed to measure the relative expression of SIRT1, PGC-1α, NRF1, Nrf2, and TFAM. (**A**) PEEK oily food migrations. (**B**) PEEK water-based food migrations. (**C**) PEEK acidic food migrations. Data are expressed as the mean ± standard deviation (*n* = 3). * *p* < 0.05, ** *p* < 0.01: compared to control cells cultured in the absence of PEEK food migrations.

It is evident that PEEK food migrations can result in oxidative damage and impair mitochondrial function. Consequently, we investigated the PGC-1α/Nrf2 signaling pathway, which plays a crucial regulatory role in chemical-induced mitochondrial damage [18]. Therefore, the PGC-1α/Nrf2 signaling pathway can be used to investigate the mechanism of mitochondria-mediated oxidative stress by exogenous chemicals [19]. These results indicated that PEEK at 2.5 mg/mL up-regulated NRF1 and Nrf2 in oily food migrations, but there was no statistical significance. However, at higher concentrations (40 mg/mL in oily and water-based food migrations; 20 mg/mL in acidic food migrations), it down-regulated the levels of SIRT1, PGC-1α, NRF1, Nrf2, and TFAM (Figure 4A–C). These results suggest that the induction of mitochondrial damage by food migrations at high PEEK concentrations may be related to the PGC-1α/Nrf2 signaling pathway.

### 3.5. PGC-1 α/Nrf2 Signaling Pathway Is Involved in PEEK Food Migrations-Induced Mitochondrial Damage

**Figure 5:**
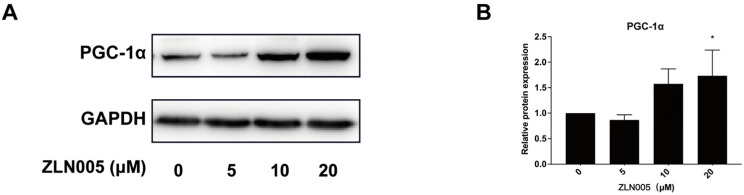
Effect of ZLN005 on PGC-1α protein expression in L-02 cells. Cells were treated with ZLN005 (0, 5, 10, and 20 μM) for 24 h. Western Blot analysis was performed to measure the relative expression of PGC-1α. (**A**) Western Blot strip map of ZLN005. (**B**) The relative expression of PGC-1α. Data are expressed as the mean ± standard deviation (*n* = 3). * *p* < 0.05: compared to control cells cultured in the absence of PEEK food migrations.

PGC-1α is a key regulator of mitochondrial biogenesis and plays an important role in oxidative stress in cells. ZLN005, a PGC-1α transcriptional activator, was chosen to investigate whether the PGC-1α/Nrf2 signaling pathway is involved in PEEK food migrations-induced mitochondrial damage. After determining the effects of different doses of PGC-1α activator ZLN005 on PGC-1α protein expression in L-02 cells, L-02 cells were treated with 20 μM ZLN005 (Figure 5A,B), food migrations at high concentrations of PEEK, and the combination of ZLN005 and PEEK food migrations for 24 h.

**Figure 6:**
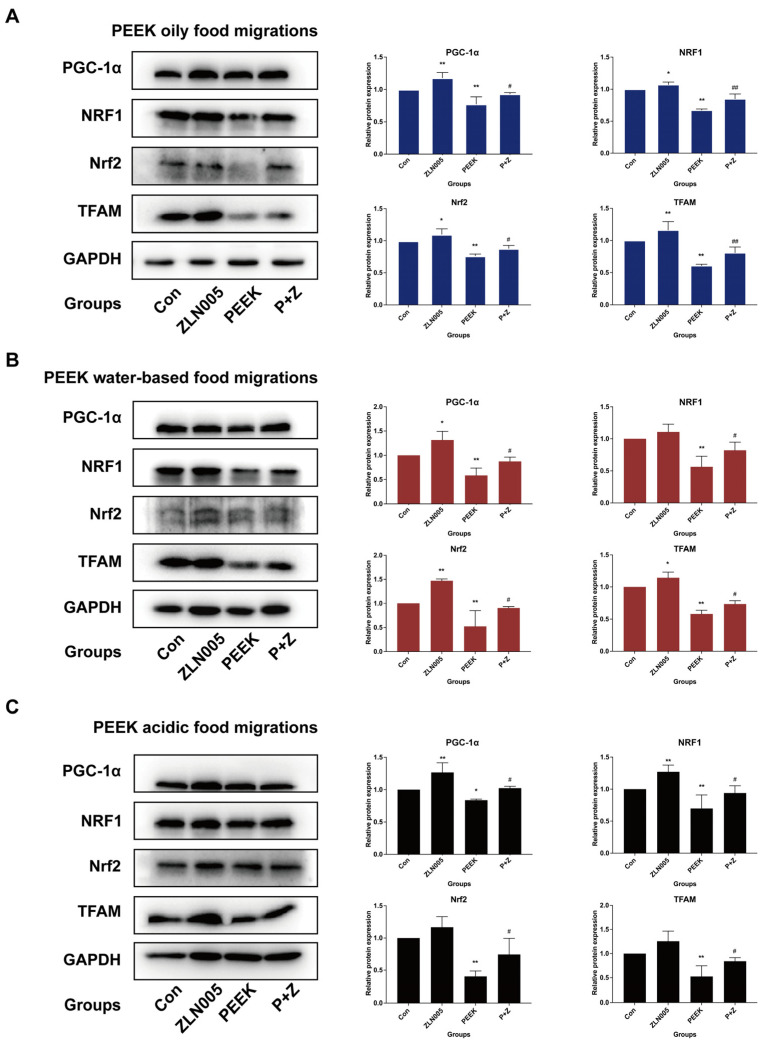
Effect of ZLN005 on the PGC-1α/Nrf2 signaling pathway inhibited by PEEK food migrations in L-02 cells. Cells were exposed to PEEK food migrations (with or without ZLN005) for 24 h. Western Blot analyses were performed to measure the relative expression of PGC-1α, NRF1, Nrf2, and TFAM. (**A**) PEEK oily food migrations. (**B**) PEEK water-based food migrations. (**C**) PEEK acidic food migrations. Data are expressed as the mean ± standard deviation (*n* = 3). * *p* < 0.05, ** *p* < 0.01: compared to the blank control cells. ^#^ *p* < 0.05, ^##^ *p* < 0.01: compared to control cells treated with PEEK food migrations alone.

The PGC-1α/Nrf2 signaling pathway protein expression levels were determined using Western Blot analysis. Compared with the blank control group, high concentrations (40 mg/mL) of PEEK oily food migrations significantly decreased the protein expression levels of PGC-1α, NRF1, Nrf2, and TFAM (*p* < 0.01). However, when co-treated with ZLN005, the protein expression levels of PGC-1α, NRF1, Nrf2, and TFAM were significantly up-regulated compared with the PEEK food migrations alone (*p* < 0.05) (Figure 6A–C). These results indicated that ZLN005 could reverse the effects of high concentrations of PEEK food migrations on the protein expression levels of key molecules in the PGC-1α/Nrf2 signaling pathway.

**Figure 7:**
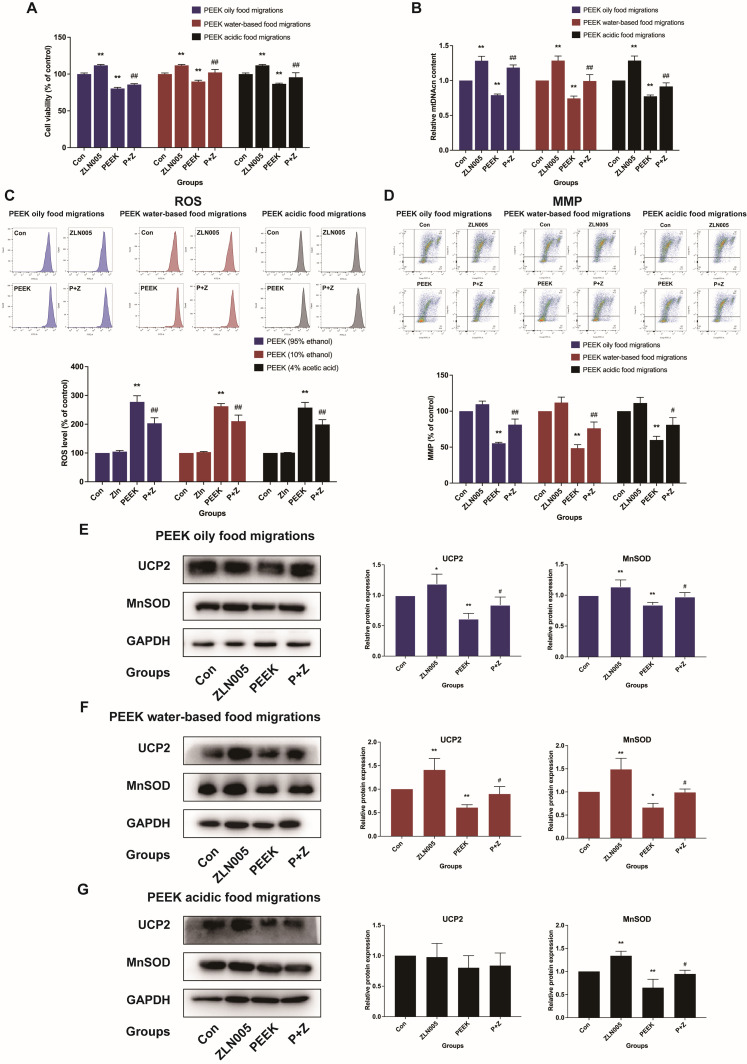
Effect of the PGC-1α/Nrf2 signaling pathway on mitochondrial function by PEEK acidic food migrations. Cells were exposed to PEEK food migrations (with or without ZLN005) for 24 h. (**A**) The viability of L-02 cells was detected by MTT assay. (**B**) The content of mtDNAcn was measured by qPCR. (**C**) ROS levels were measured with the DCFH-DA probe. (**D**) MMP levels were measured with the JC-1 probe. (**E**–**G**) The relative expression of UCP2 and MnSOD was assessed by Western Blot analysis. Data are expressed as the mean ± standard deviation (*n* = 3). * *p* < 0.05, ** *p* < 0.01: compared to the blank control cells. ^#^ *p* < 0.05, ^##^ *p* < 0.01: compared to control cells treated with PEEK food migrations alone.

To further explore the role of PGC-1α/Nrf2 signaling pathway in mitochondrial damage caused by PEEK food migrations in L-02 cells, we detected the mitochondrial damage-related indexes after treating L-02 cells with 20 μM ZLN005, PEEK food migrations, and the combination of ZLN005 and PEEK food migrations for 24 h. The result indicates that the cell viability of L-02 cells exposed to PEEK food migrations combined with ZLN005 increased significantly compared to PEEK food migrations alone (*p* < 0.05) (Figure 7A). Additionally, the co-treatment of L-02 cells with PEEK food migrations and ZLN005 notably restored mtDNAcn and MMP (*p* < 0.05) (Figure 7B,D), while significantly reducing the level of ROS (*p* < 0.05) (Figure 7C). The Western Blot analysis further revealed that the expression of UCP2 and MnSOD was significantly restored in L-02 cells co-treated with PEEK food migrations and ZLN005 together (*p* < 0.05) (Figure 7E–G). Taken together, these findings suggested that the PGC-1α/Nrf2 pathway plays an important role in the process of mitochondrial damage induced by PEEK food migrations in L-02 cells. Food migrations induce mitochondrial damage in L-02 cells at high concentrations of PEEK through the PGC-1α/Nrf2 signaling pathway.

## 4. Discussion

PEEK is an environmentally friendly and non-toxic food packaging material that has been approved by the U.S. Food and Drug Administration (FDA) for direct contact with food. It is also widely utilized in food and beverage machinery, both domestically and internationally. Research has shown that sulfonation modification of PEEK enhances excellent antimicrobial, air permeability, and moisture permeability properties, which can be used to achieve the regulation of gas composition and maintain balance within food packaging. As a result, PEEK serves as a new type of food gas-conditioning packaging material, meeting the preservation needs of diverse foods [20].

Cytotoxicity assessments, including MTT cell viability assays, alkaline phosphatase (ALP) activity measurements, and live/dead cell staining, demonstrate that PEEK exhibits no cytotoxic effects on L-929 mouse fibroblast cells and maintains normal cellular activity levels [21]. Furthermore, histopathological analysis from the systemic toxicity testing of carbon fiber-reinforced PEEK (CFRP) composites indicated no significant acute toxicity to the organisms [22]. These findings verify the material’s biological safety at both cellular and organismal levels.

In recent years, it has been found that the chemical components of food packaging materials can migrate from the surface into food during the contact process with the food, thereby entering the human body through dietary consumption and resulting in potential health risks [23]. For example, polystyrene (PS), currently a commonly used material in MAP, can produce micro-plastics that migrate into food during contact with food, leading to excessive accumulation of ROS, impaired mitochondrial function, and induced mitochondrial apoptosis and oxidative stress [24]. However, reliable toxicological data on PEEK food migrations remain scarce, and its safety profile is incompletely characterized.

Following the 2007 NRC publication of TT21C, novel in vitro toxicity-testing methods focusing on ‘toxicity pathways’ and ‘mechanisms of toxic action’ have emerged. This approach utilizes the adverse outcome pathway (AOP) framework as an innovative alternative strategy for toxicity assessment, gaining prominence in regulatory toxicology [25]. Exogenous chemicals enter the human body and produce toxic effects mainly through oxidative damage. Mitochondria are the main site of oxidative phosphorylation in vivo and the key source of intracellular ROS. Furthermore, many exogenous chemicals can directly interact with mitochondria, leading to oxidative stress, changes in mitochondrial membrane potential, and mitochondrial DNA damage, among other effects [26]. Increasingly, studies are conducting toxicity safety assessments by developing AOP frameworks that link mitochondrial damage to effects at both the organism and population levels. Analysis from AOP-Wiki and the published literature have identified cell death, oxidative stress, mitochondrial dysfunction, and lipid accumulation as the core KEs that are consistently associated with mitochondrial toxicity [27]. Consequently, we focused on mitochondrial toxicity induced by PEEK food migrations.

Preliminary findings indicate that the cell viability decreased significantly when the concentration of PEEK in oily and water-based food simulants is at 40 mg/mL, and PEEK in acidic food simulants at 20 mg/mL. Below these doses, PEEK food migrations do not significantly impact cell viability. In the preliminary stage, our research group utilized UPLC-MS/MS to analyze and identify PEEK food migrations. They found that only migrations were detected from oily food simulants, including two intended additive substances (IAS), diethyl phthalate (DEP) and 2,6-di-tert-butyl-p-benzoquinone (BHT-Q), as well as two non-intentionally added substances (NIAS), pentaethylene glycol (PEGn5) and dodecanoic acid-2-[bis (2-hydroxyethyl) amino] ethyl ester. The concentrations measured in the samples were as follows: DEP at 44.89 ng/mL, PEGn5 at 4.58 ng/mL, and dodecanoic acid-2-[2-(hydroxyethyl) amino] ethyl ester at 0.085 ng/mL, while BHT-Q exhibited a low response. A quantitative risk assessment conducted by the thresholds of toxicological concern (TTC) method revealed that the exposure levels of the above four migrations did not exceed the TTC thresholds, indicating that the PEEK material is relatively safe. While UPLC-MS/MS quantitatively detects specific migrations, it cannot evaluate downstream biological toxicity. In contrast, our mitochondrial pathway method directly links chemical migrations (validated by UPLC-MS/MS) to functional toxicity by demonstrating PEEK-induced mitochondrial dysfunction via the PGC-1α/Nrf2 signaling pathway, thereby bridging analytical chemistry with mechanistic toxicology. In conjunction with the findings of this study, it is recommended that regarding cell viability, PEEK material as a food air-conditioning packaging material is safe at low doses, and, therefore, we further examined molecular-level cellular changes at low doses.

Mitochondria serve as the principal site of ROS action, and the generation of ROS in the human body mainly depends on the aerobic metabolism of the mitochondria. Excessive ROS can disrupt intracellular redox homeostasis and induce oxidative stress. Consequently, mitochondria play a critical role in toxicity induced by exogenous compounds [28]. When mitochondria are damaged by ROS, the permeability of the mitochondrial membranes is altered, and protons are pumped from the matrix side of the inner mitochondrial membrane to the outside of the inner membrane, creating a mitochondrial transmembrane potential difference that is reduced, resulting in a decrease in the MMP and causing MMP depolarization [29]. Mitochondrial DNA and respiratory chain complexes, etc., are damaged, contributing to the cell being in a state of oxidative stress and resulting in organ damage [30]. DEP is a plasticizer used in food packaging materials, which can cause redox imbalance and oxidative damage in organisms, accumulating in the liver, causing hepatotoxicity [31]. Meanwhile, BHT-Q has been shown to generate superoxide radicals in aqueous solutions, which can indirectly damage DNA and cause DNA cleavage by producing H_2_O_2_ at a concentration as low as 1 × 10^−6^ M [32]. We therefore assessed mitochondrial damage as an indicator of PEEK food migration toxicity.

Electron microscopy revealed mitochondrial structural damage after 24 h exposure to PEEK food migrations. The results show that PEEK food migrations damage mitochondrial structure and increase intracellular ROS levels depending on the dose of PEEK in oily, water-based, and acidic food simulants. Furthermore, PEEK oily and water-based food migrations could decrease ATP levels, while acidic food migrations at different PEEK concentrations elevated cellular ATP levels. This opposite response may reflect different cellular mechanisms. The ATP elevation observed with acidic migrants could represent a compensatory response to mild mitochondrial stress, temporarily enhancing energy production to counteract oxidative damage [33]. In contrast, the ATP reduction caused by oily and water-based migrants likely reflects mitochondrial dysfunction and impaired energy metabolism under higher cytotoxic stress [34]. Alternatively, the differing ATP responses may be a plausible hypothesis given possible compositional differences among food simulants, although direct evidence is currently lacking. Specifically, 2.5–40 mg/mL of PEEK in oily and water-based food simulants, as well as 20 mg/mL of PEEK in acidic food simulants, could decrease mtDNAcn. Additionally, MMP exhibited a biphasic response, initially rising and then falling. Moreover, the expression of UCP2 and MnSOD was down-regulated after treatment of PEEK food migrations, which are related to mitochondrial levels [35]. Overall, these findings suggest that food migrations may increase or slightly decrease the indicators of mitochondrial function at low concentrations of PEEK without statistical significance, which could be a compensatory response. This suggests PEEK at high concentrations may produce mitochondrial toxicity through the oxidative stress pathways when used as food air-conditioning packaging materials.

The mtDNAcn are the key indicators of mitochondrial biogenesis [36]. The overproduction of ROS is associated with mitochondrial structure and function damage. Mitochondrial biogenesis is regarded as a compensatory response to restore mitochondrial components and maintain homeostasis [37]. To investigate whether mitochondrial function injury and mtDNAcn reductions result from impaired biogenesis, we focused on the PGC-1α/Nrf2 pathway. We measured the levels of SIRT1, PGC-1α, NRF1, Nrf2, and TFAM in cells using Western Blot analysis. PGC-1α, a master regulator of mitochondrial biogenesis, may lead to a decrease in mtDNAcn [38]. Yi Li et al. found that Nrf2 collaborates with PGC-1α to regulate mitochondrial gene transcription, influencing the expression of NRF1 and TFAM during the process of mitochondrial biogenesis [14]. Nrf2 is known to scavenge excess ROS to protect cells from oxidative stress injury and maintain redox homeostasis. Elevated ROS levels stimulate Nrf2 signaling [39]. The Nrf2 signaling pathway is a crucial host defense pathway, as a major mediator of antioxidant transcription [40,41]. SIRT1 plays a vital role in regulating cellular responses to oxidative stress, activating the PGC-1α-mediated transcription of mitochondrial genes involved in mitochondria proliferation, oxidative phosphorylation, and energy production [42]. Food migrations could decrease mtDNAcn and MMP, increase ROS levels, and down-regulate the expression of UCP2 and MnSOD significantly in L-02 cells at high concentrations of PEEK, thus causing mitochondrial damage. Our study showed that after the treatment of PEEK food migrations for 24 h, oily, water-based, and acidic food migrations could reduce the expression of SIRT1, PGC-1α, NRF1, and Nrf2 at high PEEK doses. The effect of the PGC-1α/Nrf2 signaling pathway suggested that the PGC-1α/Nrf2 pathway may be implicated in the process of mitochondrial damage induced by PEEK food migrations in L-02 cells.

To further investigate the relationship between the PGC-1α/Nrf2 signaling pathway and mitochondrial damage induced by PEEK food migrations, we co-treated L-02 cells with PEEK food migrations and ZLN005. The protein expression levels of key molecules in the PGC-1α/Nrf2 pathway was assessed using Western Blot analysis, and the results indicated that ZLN005 effectively reversed the impact of high concentrations of PEEK food migrations on the protein expression levels of PGC-1α/Nrf2 pathway molecules. Subsequently, we detected cell viability, mtDNAcn, ROS, and MMP levels, the protein expression levels of UCP2 and MnSOD in L-02 cells treated with 20 μM ZLN005, and a high concentration of PEEK food migrations. The results showed that, compared to PEEK food migrations alone, co-treatment with ZLN005 significantly increased viability, mtDNAcn, MMP, and the protein expression levels of UCP2 and MnSOD, while ROS levels were notably reduced. These findings suggest that by upregulating the PGC-1α pathway, the level of intracellular oxidative stress can be reduced, mitochondria can be protected, and cell survival can be improved, thus alleviating the cellular damage caused by food migrations at high concentrations of PEEK. Collectively, high concentrations of PEEK food migrations induce mitochondrial damage via the PGC-1α/Nrf2 signaling pathway in L-02 cells.

The safety of PEEK remains uncertain due to the lack of reliable toxicological data. This study is the first to investigate the toxicity of PEEK materials through in vitro cellular experiments and to study the mitochondrial damage effects of migrations of PEEK food packaging materials based on the harmful outcome pathway, so as to provide a scientific basis and technical guarantee for the subsequent risk assessment and safe use of PEEK-modified atmosphere packaging. This study was conducted in accordance with national standards using packaging materials migrations from various types of food that are the substances humans are actually exposed to, providing a scientific basis for the hazard assessment of PEEK as a food packaging material. Therefore, we offer novel hazard assessment data for PEEK food migrations.

It is important to acknowledge certain limitations of this study. We selected only the L-02 cell line to construct the mitochondrial AOP framework, in view of the different sensitivities of different cells to exogenous chemicals. Further validation with other cell lines or liver organoids is necessary in the future. Additionally, this study did not consider the metabolic activation of PEEK food migrations within the human body. Further research should explore whether metabolic activation occurs in the human body after entering the body, and whether its toxicity changes before and after metabolic activation, thereby providing a more comprehensive evaluation of the safety of PEEK materials. Finally, provided the condition of ensuring the safety of PEEK migration products, whether the additives are toxic is also an area of research that our research group will carry out.

## 5. Conclusions

In summary, food migrations at low concentrations of PEEK do not significantly affect cell viability in L-02 cells. However, at a PEEK concentration of 40 mg/mL, oily and water-based food migrations can cause cell damage, while at a PEEK concentration of 20 mg/mL, acidic food migrations lead to cell damage. Both oily and water-based food migrations induced mitochondrial toxicity in L-02 cells at PEEK concentrations up to 40 mg/mL, whereas acidic food migrations caused mitochondrial toxicity at PEEK concentrations up to 20 mg/mL. The PGC-1α/Nrf2 pathway plays a key role in this process. This study underscores that a safe dosage of PEEK should be considered when utilizing this material for food packaging purposes.

## Data Availability

The data that support the findings of this study are available on request from the corresponding author. The data are not publicly available due to privacy or ethical restrictions.

## Supplementary Materials

The following supporting information can be downloaded at https://www.mdpi.com/article/10.3390/toxics13121054/s1; Table S1: PCR Primer Sequences; Figure S1: Genetic authentication of L-02 cells; Text S1: PEEK Migration Test; Text S2: Row Experimental Record; Table S2: Migration Test for PEEK Food Contact Material; Text S3: Batch Consistency Verification by UPLC-MS/MS; Text S4: Identification of Migrations from PEEK; Figure S2: Positive ion mode (ESI+) and negative ion mode (ESI−) base peak plots of PEEK migrants in different food simulants.

## Abbreviations

The following abbreviations are used in this manuscript:

PEEK	Poly Ether-Ether Ketone
MAP	Modified atmosphere packaging
ROS	Reactive oxygen species
MMP	Mitochondrial membrane potential
mtDNAcn	Mitochondrial DNA copy number
AOP	Adverse Outcome Pathway
MIE	Molecular initiating event
AO	Adverse Outcome
KEs	Key events
