# Supplementary material for: Effect of Food Migrations of PEEK-Modified Atmosphere Packaging Materials on Mitochondrial Damage via PGC-1α/Nrf2 Signaling Pathway
Source: Toxics. 2025 Dec 5;13(12):1054. doi: 10.3390/toxics13121054 (PMC12737303; doi:10.3390/toxics13121054)
Supplement: Supplementary file 1 [file toxics-13-01054-s001.zip › toxics-3995170-supplementary.pdf]

Supplementary Materials

Sihui Guo, Kaile Li, Wei Li, Hao Huang, Yalan Zhang, Qinwen Zhou, Qi He, Zhini He, Weiliang Wu, Xingfen Yang * and Qinzhi Wei *

Food Safety and Health Research Center, Guangdong Provincial Key Laboratory of Tropical Disease Research, School of Public Health, Southern Medical University, Guangzhou 510515, China; gsh79190624@163.com (S.G.); lkl5454han@163.com (K.L.); lwliweiyy@163.com (W.L.); hh4240123@163.com (H.H.); zhangyalan0616@163.com (Y.Z.); qwzhouz@163.com (Q.Z.); heqi@163.com (Q.H.); hezhinihzn@126.com (Z.H.); wu1108@smu.edu.cn (W.W.)

* Correspondence: yangalice79@smu.edu.cn (X.Y.); cnwei99@smu.edu.cn (Q.W.)

Supplementary Table S1. PCR Primer Sequences

Target Gene	Primer sequence (5'-3')-Forward	Primer sequence (5'-3')-Reverse
ND1	CCCTAAAACCCGCCACATCT	GAGCGATGGTGAGAGCTAAGGT
HBG	GCTTCTGACACAACTGTGTTCACTAGC	CACCAACTTCATCCACGTTCACC

A

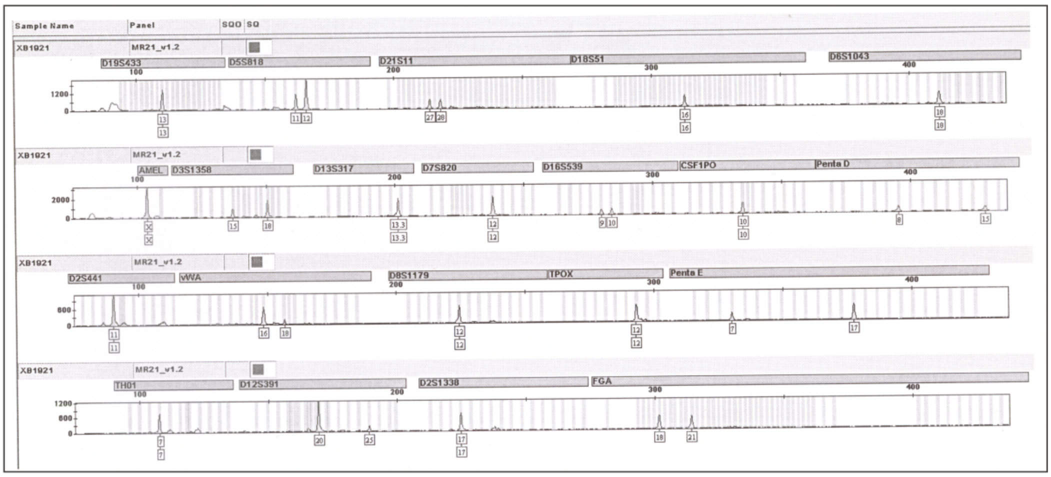

B

Table 1. The alleles of 21 locations in HL-7702 cell line

HL-7702 cell line（Fig. No.XB1921）		
Marker	Allele 1	Allele 2
D19S433	13	13
D5S818	11	12
D21S11	27	28
D18S51	16	16
D6S1043	18	18
AMEL	X	X
D3S1358	15	18
D13S317	13.3	13.3
D7S820	12	12
D16S539	9	10
CSF1PO	10	10
Penta D	8	15
D2S441	11	11
vWA	16	18
D8S1179	12	12
TPOX	12	12
Penta E	7	17
TH01	7	7
D12S391	20	25
D2S1338	17	17
FGA	18	21

C

中国典型培养物保藏中心

CHINA CENTER FOR TYPE CULTURE COLLECTION (CCTCC)

Wuhan University, Wuhan 430072, China

Phone: 86-027-68752093 **Fax:** 86-027-68754833 **Email:** shenchao@whu.edu.cn

06-16-2014

Entrusted by Laboratory of Nanjing Medical University, CCTCC has conducted identification experiments on the HL-7702 cell line, and come to the following conclusions:

1. There was no third allele found in all the locations of HL-7702 cell line, it indicating that there was no cross-contaminant of human source cell line.
2. Compared the STR data of HL-7702 cell line in the databases of ATCC, JCRB and DSMZ, its profile does not exactly match with any of the current data (Table 1).

Director's Signature:

China Center for Type Culture Collection

Supplementary Figure S1. Genetic authentication of L-02 cells. L-02 cells, also called HL-7702 cells, were characterized by the China Center for Type Culture Collection (CCTCC, Wuhan University, Wuhan, China) using short tandem repeat (STR) analysis. (A) Gene map of STR markers in L-02 cells, and (B) listed in a table. (C) Authentication report of L-02 cells by the CCTCC.

Text S1. PEEK Migration Test

1.1 Materials and Reagents

PEEK was purchased from Shanghai Yingcai Industrial Co., Ltd (Shanghai, China). Anhydrous ethanol (HPLC) and acetic acid (HPLC) were purchased from Sigma-Aldrich (St. Louis, MO, USA). All graduated cylinders, beakers, conical flasks, and volumetric flasks were purchased from Shubo Group (Sichuani, China)), while the 30mL amber sample vials were sourced from Bikeman Biotechnology (Changdei, China)).

1.2 Preparation of Food Simulants

According to GB 31604.1-2015 National Food Safety Standard - General Rules for Migration Testing of Food Contact Materials and Articles and the intended use of the PEEK film, three food simulants were selected: 95% (v/v) ethanol to simulate fatty foods, 10% (v/v) ethanol to simulate aqueous foods, and 4% (v/v) acetic acid to simulate acidic foods. The preparation methods are as follows:

95% (v/v) ethanol: Clean a 100 mL measuring cylinder. Add 50 mL of ultrapure water and transfer to a volumetric flask. Dilute to 1 L with ethanol. Used to simulate fatty foods.

10% (v/v) ethanol: Clean a 100 mL measuring cylinder. Add 100 mL of ethanol and transfer to a volumetric flask. Dilute to 1 L with ultrapure water. Used to simulate aqueous foods.

4% (v/v) acetic acid: Clean a 50 mL measuring cylinder. Add 40 mL of acetic acid and transfer to a volumetric flask. Dilute to 1 L with ultrapure water. Used to simulate acidic foods.

1.3 Film Cutting and Weighing

Cut the PEEK film into pieces measuring 30 cm^2 (5 cm × 6 cm). Wash the pieces 10 times with ultrapure water, dry in an oven for 30 minutes, weigh on the balance, and record the weights.

1.4 Migration Test

Perform the migration test according to GB 31604.1-2015 National Food Safety Standard - General Rules for Migration Testing of Food Contact Materials and Articles. Place five pieces of the 30 cm^2 PEEK film into a 250 mL glass conical flask containing the food simulant, ensuring the films are completely immersed. Considering the expected contact time and temperature with food, place the flask in a constant temperature water bath (Shanghai Yiheng Instruments DK-8D, China) maintained at 40°C for 10 days to conduct the migration test.

1.5 Concentration and Reconstitution

After migration, transfer the migration solution to a rotary evaporator (IKA Works RV 10, Germany) and concentrate to approximately 10 mL at 50°C. Further dry the concentrate using a nitrogen evaporator (Beijing Hengao Tech HAD-24, China) at 55°C.. Finally, reconstitute the residue in DMSO to prepare a stock solution of PEEK food migrations with a concentration of 4000 mg/mL for subsequent experiments.

Text S2. Row Experimental Record

Three discrete migration tests were performed using PEEK material under standardized conditions: for fatty food simulation with 95% (v/v) ethanol (PEEK mass: 9.3759 g), aqueous food simulation with 10% (v/v) ethanol (9.3755 g), and acidic food simulation with 4% (v/v) acetic acid (9.2466 g). All tests employed identical processing parameters，3000 mL migration volume concentrated and reconstituted to 2 mL final volume (**Supplementary Table S2**).

Concentration of the final reconstituted solution can be calculated as:

$$\text{Concentration} = \frac{\text{PEEK mass (g)} \times 1000}{\text{Reconsititution volume (mL)}} \ \text{(units: mg/mL)}$$

Supplementary Table S2. Migration Test for PEEK Food Contact Material

Mass of PEEK (g)	Food Type	Food Simulants	Migration Volume (mL)	Reconstitution Volume (mL)
9.3759	Fatty foods	95% (v/v) ethanol	3000	2
9.3755	Aqueous foods	10% (v/v) ethanol	3000	2
9.2466	Acidic foods	4% (v/v) acetic acid	3000	2

Text S3. Batch Consistency Verification by UPLC-MS/MS

UPLC-MS/MS analysis confirmed batch consistency using an amide column with a mobile phase of 0.1% aqueous formic acid + 5 mmol/L ammonium acetate (Phase A) and acetonitrile (Phase B) at 0.20 mL/min flow rate, maintaining the autosampler tray at 5°C with 2 μL injection volume. Mass spectrometric detection employed ESI+ and ESI- ionization modes with Full MS/ddMS^2 acquisition scanning m/z 100-1200 (selected as substances >1000 Da are generally non-absorbable), achieving 70,000 FWHM resolution at m/z 200. Optimized parameters included a 3.5 kV HESI III spray voltage and 400°C capillary temperature, with system calibration performed using Pierce LTQ Velos ESI calibration solutions.

Text S4. Identification of Migrations from PEEK

Analysis of the base peak chromatograms obtained from UPLC-MS/MS characterization of PEEK food migrants revealed consistent compositional profiles across different batches. Consequently, bulk quantities of migrants derived from fatty, aqueous, and acidic food simulants were prepared under identical conditions to facilitate subsequent in vitro cellular experiments.

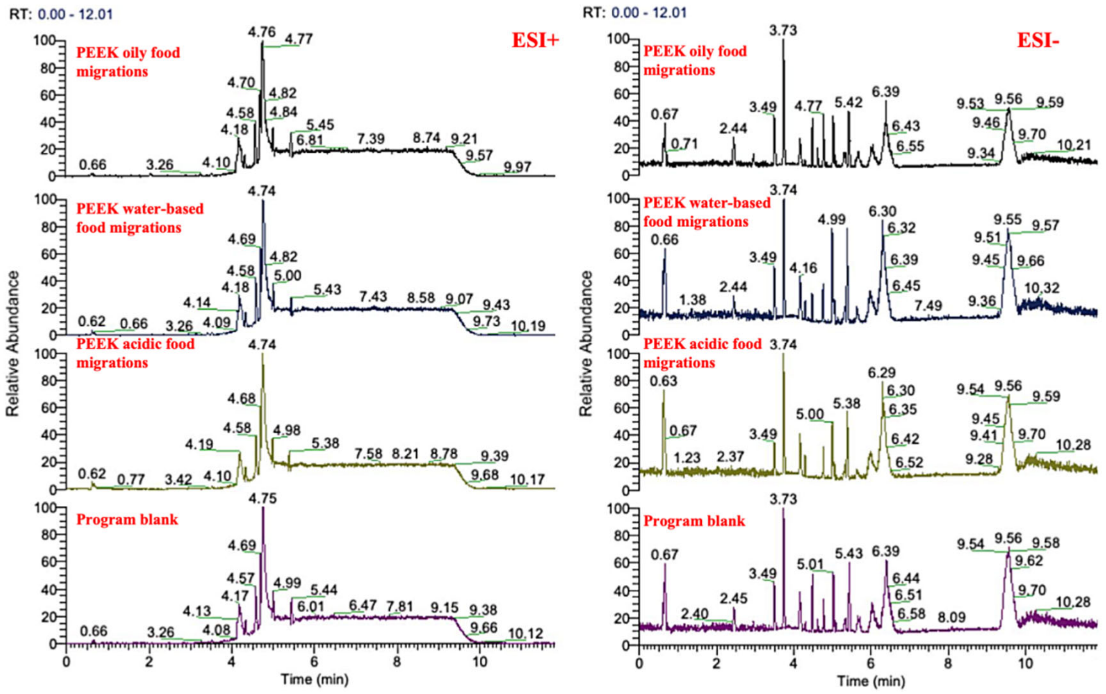

Supplementary Figure S2. Positive ion mode (ESI+) and negative ion mode (ESI-) base peak plots of PEEK migrants in different food simulants.
